# Outcomes of mechanical thrombectomy in orally anticoagulated patients with anterior circulation large vessel occlusion: a propensity-matched analysis of the Imperial College Thrombectomy Registry

**DOI:** 10.1007/s00415-023-11926-5

**Published:** 2023-08-18

**Authors:** Lucio D’Anna, Michele Romoli, Matteo Foschi, Samir Abu-Rumeileh, Tsering Dolkar, Orsolya Vittay, Luke Dixon, Paul Bentley, Zoe Brown, Charles Hall, Sohaa Jamil, Harri Jenkins, Joseph Kwan, Roberta La Cava, Maneesh Patel, Neil Rane, Dylan Roi, Abhinav Singh, Marius Venter, Omid Halse, Abid Malik, Dheeraj Kalladka, Soma Banerjee, Kyriakos Lobotesis

**Affiliations:** 1 Department of Stroke and Neuroscience, Charing Cross Hospital, Imperial College London NHS Healthcare Trust, London, UK; 2 Department of Brain Sciences, Imperial College London, London, UK; 3 Neurology and Stroke Unit, Department of Neuroscience, Bufalini Hospital, AUSL Romagna, Cesena, Italy; 4 Department of Biotechnological and Applied Clinical Sciences, University of L’Aquila, L’Aquila, Italy; 5 Department of Neurology, Martin-Luther-University Halle-Wittenberg, Halle (Saale), Germany; 6 Neuroradiology, Department of Imaging, Charing Cross Hospital, Imperial College London, NHS Healthcare Trust, London, UK

**Keywords:** Mechanical thrombectomy, Oral anticoagulants, Ischemic stroke, Large vessel occlusion

## Abstract

**Background:**

Mechanical thrombectomy (MT) remains an effective treatment for patients with acute ischemic stroke receiving oral anticoagulation (OAC) and large vessel occlusion (LVO). However, to date, it remains unclear whether MT is safe in patients on treatment with OAC.

**Aims:**

In our study, we performed a propensity-matched analysis to investigate the safety and efficacy of MT in patients with acute ischemic stroke receiving anticoagulants. A propensity score method was used to target the causal inference of the observational study design.

**Methods:**

This observational, prospective, single-centre study included consecutive patients with acute LVO ischemic stroke of the anterior circulation. Demographic, neuro-imaging and clinical data were collected and compared according to the anticoagulation status at baseline, patients on OAC vs those not on OAC. The primary study outcomes were the occurrence of any intracerebral haemorrhage (ICH) and symptomatic ICH. The secondary study outcomes were functional independence at 90 days after stroke (defined as modified Rankin Scale (mRS) scores of 0 through 2), mortality at 3 months and successful reperfusion rate according to the modified treatment in cerebral infarction (mTICI) score.

**Results:**

Overall, our cohort included 573 patients with acute ischemic stroke and LVO treated with MT. After propensity score matching, 495 patients were matched (99 OAC group vs 396 no OAC group). There were no differences in terms of clinical characteristics between the two groups, except for the rate of intravenous thrombolysis less frequently given in the OAC group. There was no significant difference in terms of the rate of any ICH and symptomatic ICH between the two groups. With regards to the secondary study outcome, there was no significant difference in terms of the rate of successful recanalization post-procedure and functional independence at 3 months between the two groups. Patients in the OAC group showed a reduced mortality rate at 90 days compared to the patients with no previous use of anticoagulation (20.2% vs 21.2%, *p* = 0.031). Logistic regression analysis did not reveal a statistically significant influence of the anticoagulation status on the likelihood of any ICH (OR = 0.95, 95% CI = 0.46–1.97, *p* = 0.900) and symptomatic ICH (OR = 4.87, 95% CI = 0.64–37.1, *p* = 0.127). Our analysis showed also that pre-admission anticoagulant use was not associated with functional independence at 90 days after stroke (OR = 0.76, 95% CI = 0.39–1.48, *p* = 0.422) and rate of successful reperfusion (OR = 0.81, 95% CI = 0.38–1.72, *p* = 0.582).

**Conclusion:**

According to our findings anticoagulation status at baseline did not raise any suggestion of safety and efficacy concerns when MT treatment is provided according to the standard guidelines. Confirmation of these results in larger controlled prospective cohorts is necessary.

**Supplementary Information:**

The online version contains supplementary material available at 10.1007/s00415-023-11926-5.

## Introduction

Mechanical thrombectomy (MT) remains an effective treatment for patients with acute ischemic stroke receiving oral anticoagulation (OAC) and large vessel occlusion (LVO) [1]. However, to date, it has been a matter of controversy if MT in acute ischemic stroke patients with prior anticoagulant treatment carries a major risk of bleeding. Indeed, patients taking OAC were often underrepresented in pivotal clinical trials of MT, accounting for < 5% of the total [2]. Therefore, data regarding the safety of MT in anticoagulated patients rely mainly on observational retrospective studies with the major limitation of the lack of adjustment for confounding factors [3–13]. Then, it remains unclear whether MT is safe in patients on treatment with OAC.

## Aims

In this present study we performed a propensity-matched analysis to investigate the safety and efficacy of MT in patients with acute ischemic stroke receiving anticoagulants. We opted to perform a propensity score method because it aims to target causal inference in observational studies in a similar manner to randomised studies by facilitating the measurement of differences in outcomes between the treated population and a reference population.

## Methods

In this observational, investigator-initiated, prospective study, all acute stroke patients consecutively treated with MT at the Stroke Department, Charing Cross Hospital, Imperial College Healthcare NHS Trust, London between 1st January 2016 and 30th June 2021 were included. This study has obtained approval from the UK Health Regulator Authority (HRA) (HRA Reference No.: 275260). The study has also received confirmation of capacity and capability from the Imperial College Healthcare NHS Trust. The study was conducted in accordance with the recommendations for physicians involved in research on human subjects adopted by the 18th World Medical Assembly, Helsinki 1964 and later revisions. The Stroke Department at Charing Cross Hospital is the Northwest London (UK) regional Comprehensive Stroke Center (CSC) for MT in an urban metropolitan area with more than 6.4 million people. Please refer to our previous manuscripts for the organization of the Imperial Stroke Thrombectomy network [14, 15].

### Patient inclusion and exclusion criteria for the analysis

For the purpose of this analysis, the criteria for patients selection were: (1) age ≥ 18 years; (2) NIHSS score 6 or more; (3) Alberta Stroke Program Early CT score (ASPECTS) [16] 5 or more; (4) LVO sites: distal internal carotid artery, middle cerebral artery segments M1 or M2; (5) initiation of the MT had to be possible within 6 h after the stroke onset. IVT was administered in all patients who presented within 4.5 h of stroke symptom onset without contraindications according to the guidelines. For this analysis, we excluded stroke patients with basilar artery occlusion and patients that met DAWN or DEFUSE three eligibility criteria [17, 18].

### Clinical and radiological assessments

The Imperial Stroke Centre registry prospectively collected data from consecutive patients treated with MT and encompassed patient characteristics, including age, vascular risk factors, laboratory results, relevant medical history. The prescription of any anticoagulant before admission was recorded. Direct oral anticoagulant (DOAC) therapy was defined as one of the following drugs and dosages: apixaban 2.5 mg or 5 mg twice daily; dabigatran 110 mg or 150 mg twice daily; edoxaban 30 mg or 60 mg once daily; or rivaroxaban 15 mg or 20 mg once daily. Vitamin K antagonist (VKA) was defined as treatment with acenocoumarol/warfarin. The choice of treatment was decided by the treating physician as part of routine clinical care pre-admission. For the purpose of this analysis, we excluded patients reported to be non-complaint with the treatment. NIHSS was performed in all patients on admission and 24 h after the MT. The modified Rankin Scale (mRS) was used to assess the patient’s initial pre-stroke status and the level of functional independence at 90 days of the patients was evaluated centrally through a telemedicine consultation or in-person consultation. Procedural metrics were collected prospectively. The extent of the initial core infarct was determined on pre-therapeutic CT using ASPECTS [16]. In addition, an independent rater (consultant neuroradiologist) who did not participate in the endovascular stroke treatment of included patients, evaluated pre-therapeutic CT, and follow-up CT at 24 h.

### Study endpoints

The primary study outcome measures were the occurrence of any intracerebral haemorrhage (ICH) and symptomatic ICH (sICH; according to Safe Implementation of Thrombolysis in Stroke-Monitoring Study [SITS-MOST], European Cooperative Acute Stroke Study-II [ECASS-II], Solitaire With the Intention for Thrombectomy as Primary Endovascular Treatment [SWIFT-PRIME]. The secondary study outcomes were functional independence at 90 days after stroke (defined as modified Rankin Scale (mRS) scores of 0 through 2), mortality at 3 months and successful reperfusion rate according to the modified treatment in cerebral infarction (mTICI) score.

### Statistical analysis

Categorical variables are presented as count and percentage, continuous variables as mean and standard deviation or median and interquartile range according to normal distribution. Comparisons were considered significant at *p* < 0.05. A propensity-score (PS) matching algorithm was implemented to mitigate potential differences across people receiving anticoagulation vs controls in age, gender, hypertension, previous stroke and CHA_2_DS_2_VASc score. PS of the treatment variable (anticoagulation vs none) was calculated for each patient and a 1:4 nearest neighbour matching no-replacing algorithm was used to match patients on anticoagulation to patients not on anticoagulation within 0.25 × SD of the logit of the propensity score according to their status. To determine whether the propensity score approach achieved balance in all potential confounders, we compared all baseline characteristics between the two groups. Statistical comparisons were performed between patient subgroups using the *χ*^2^ test, Fisher exact test, Student *t* test, and Mann–Whitney *U* as indicated for dichotomous or continuous variables. Backward stepwise binary logistic regression was implemented to weight the predictive value of anticoagulation at baseline on predefined outcomes. Logistic regression was modelled for the primary outcome and secondary outcomes depending on factors emerging from univariate analysis, with matched variables and anticoagulation status implemented a priori. Statistical analysis was performed with R version 3.3.1.

## Results

**Fig. 1:**
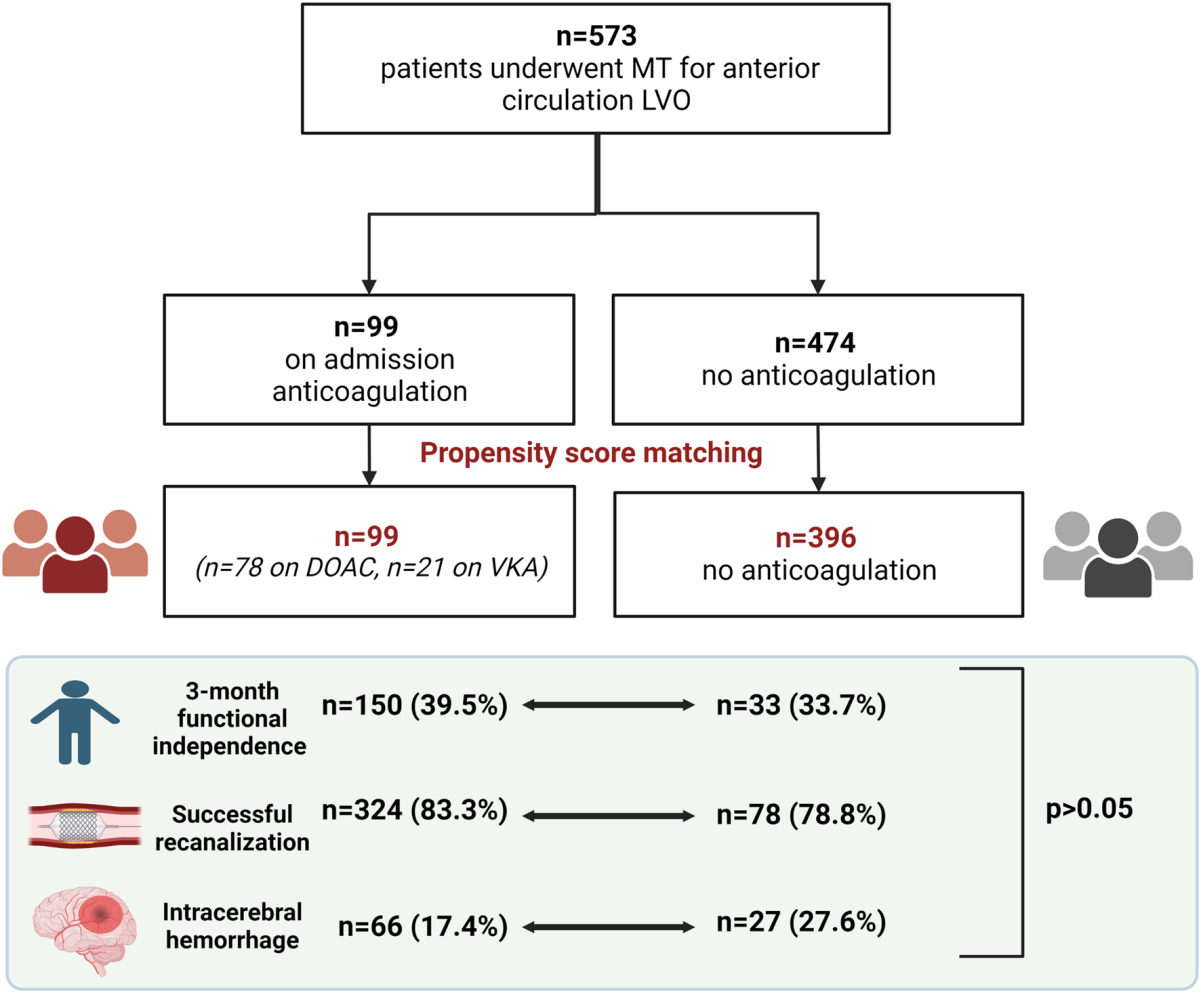
Study algorithm

During the study period, 573 patients with acute ischemic stroke and LVO of the anterior circulation underwent MT in our centre (Fig. 1). There were 99 patients out of 573 (17.3%) with anticoagulation use pre-admission while there were 474 (82.7%) patients with no previous anticoagulation use. In the anticoagulation group, 89 out of 99 (89.9%) were on treatment for atrial fibrillation, 3 out of 99 (3%) for the presence of a metallic valve, 4 out of 99 (4%) for deep venous thrombosis, 1 out of 99 (1%) for pulmonary embolism and 1 out of 99 (1%) for peripheral vascular disease. Regarding the baseline characteristics according to the anticoagulation status (Supplementary Table 1), patients on anticoagulation were older, had a higher CHA_2_DS_2_VASc score, more often hypertension and a previous ischemic stroke. As expected, intravenous thrombolysis was less frequently given in the anticoagulation group.

After propensity score matching, 495 patients were matched. There were 99 patients in the anticoagulation group (78 on DOAC and 21 on VKA) and 396 patients with no previous anticoagulation use (Fig. 1). There were no differences in terms of clinical characteristics between the two groups, except for the rate of intravenous thrombolysis less frequently given in the anticoagulation group (Table 1). We did not observe any significant differences between the two groups in regards to the MT techniques used (Supplemental Table 2). In terms of the primary study outcome, there was no significant difference in terms of rate of any ICH and symptomatic ICH between the two groups. With regards to the secondary study outcome, there was no significant difference in terms of rate of successful recanalization post-procedure and functional independence at 3 months between the two groups. Patients in the anticoagulation group showed a reduced mortality rate at 90 days compared to the patients with no previous use of anticoagulation (20.2% vs 21.2%, *p* = 0.031) (Table 2).

**Table 1:** Characteristics of the two cohorts of patients

	No anticoagulation (*n* = 396)	Anticoagulation (*n* = 99)
Age, year, (Mean, sd)	71.9 ± 13.1	72 ± 11.1
CHA2DS2VASc (Median, IQR)	5 (5–6)	5 (5–6)
Female, *n* (%)	180 (45.5%)	52 (52.5%)
Hypertension, *n* (%)	232 (58.6%)	65 (65.7%)
Diabetes, *n* (%)	88 (22.2%)	21 (21.2%)
Dyslipidemia, *n* (%)	199 (50.3%)	46 (46.5%)
Coronary artery disease, *n* (%)	75 (18.9%)	19 (19.2%)
Non-smoking, *n* (%)	317 (80.1%)	83 (83.8%)
Previous ischemic stroke, *n* (%)	30 (7.6%)	13 (13.1%)
Malignancy, *n* (%)	34 (8.6%)	13 (13.1%)
Dementia, *n* (%)	4 (1%)	0 (0%)
mRS 0–2 at baseline, *n* (%)	396 (100%)	99 (100%)
Platelets (× 10^9^/L), (Mean, sd)	235.7 ± 163.6	221.6 ± 94.5
aPTT (Mean, sd)	30.6 ± 16.6	35.4 ± 31.6*
Creatinine, (Mean, sd)	83.4 ± 57.7	82.9 ± 28.4
Systolic Blood Pressure on admission, (Mean, sd)	146 ± 24.4	147 ± 26.5
Diastolic Blood Pressure on admission, (Mean, sd)	81 ± 15.7	82 ± 16.3
Thrombolysis, *n* (%)	328 (82.8%)	22 (22.2%)***
NIHSS at baseline, (Median, IQR)	18 (18–19)	18 (17–20)
Onset to needle (min), (Mean, sd)	129 ± 55.5	136 ± 56.9
Door to needle time (min), (Mean, sd)	42 ± 29.9	55 ± 42
Onset to groin puncture time (min), (Mean, sd)	277 ± 86.9	297 ± 170.4
ASPECTS, (Median, IQR)	8 ± 2	8 ± 1

*mRS* modified Rankin Scale,* NIHSS* National Institutes of Health Stroke Scale,* ASPECTS* Alberta Stroke Program Early CT score,* sd* standard deviation,* IQR* interquartile range

*p < 0.05, **p < 0.01, ***p < 0.001

**Table 2:** Outcome distribution in the two cohorts of patients

	No anticoagulation (*n* = 396)	Anticoagulation (*n* = 99)
mRS 0–2 at 90 days	150 (39.5%)	33 (33.7%)
Successful recanalization (mTICI 2b-3)	324 (83.3%)	78 (78.8%)
sICH	20 (5.1%)	1 (1%)
Intracerebral haemorrhage	66 (17.4%)	27 (27.6%)
Death	84 (21.2%)	20 (20.2%)*

*mRS* modified Rankin Scale,* ICH* Intracebral Haemorrhage,* TICI* modified thrombolysis in cerebral infarction classification

^*^p < 0.05, **p < 0.01, ***p < 0.001

Logistic regression analysis did not reveal a statistically significant influence of the anticoagulation status on the likelihood of any ICH (OR = 0.95, 95% CI = 0.46–1.97, *p* = 0.900) and symptomatic ICH (OR = 4.87, 95% CI = 0.64–37.1, *p* = 0.127) (Table 3). Our analysis showed also that pre-admission anticoagulant use was not associated with functional independence at 90 days after stroke (OR = 0.76, 95% CI = 0.39–1.48, *p* = 0.422) (Table 4) and rate of successful reperfusion (OR = 0.81, 95% CI = 0.38–1.72, *p* = 0.582) (Table 5).

**Table 3:** Regression modelling for predictors of symptomatic and any intracerebral haemorrhage

	OR (95% CI)	Elimination step	*p* value
Symptomatic intracerebral haemorrhage			
ASPECT score	0.78 (0.6–1.01)	/	0.057
Systolic Blood Pressure on admission	1.02 (1–1.04)	/	0.042
Anticoagulation	4.87 (0.64–37.1)	/	0.127
Thrombolysis	0.99 (0.31–3.14)	1	0.986
Hypertension	1.11 (0.32–3.83)	2	0.874
NIHSS on admission	0.99 (0.91–1.08)	3	0.818
Previous ischemic stroke	0.46 (0.12–1.74)	4	0.251
Age	0.98 (0.93–1.02)	5	0.289
CHA2DS2VASc	1.16 (0.81–1.67)	6	0.415
Female	0.58 (0.23–1.49)	7	0.255
Any intracerebral haemorrhage			
ASPECT score	0.69 (0.59–0.8)	/	< 0.001
NIHSS on admission	1.06 (1.01–1.11)	/	0.018
Female	0.6 (0.37–0.97)	/	0.037
Hypertension	0.54 (0.32–0.9)	/	0.019
CHA2DS2VASc	1.01 (0.75–1.36)	1	0.933
Anticoagulation	0.95 (0.46–1.97)	2	0.900
Thrombolysis	0.91 (0.53–1.55)	3	0.724
Systolic Blood Pressure on admission	1 (0.99–1)	4	0.326
Previous ischemic stroke	0.61 (0.28–1.35)	5	0.225
Age	0.99 (0.97–1)	6	0.129

*NIHSS* National Institutes of Health Stroke Scale, *ASPECTS* Alberta Stroke Program Early CT score

**Table 4:** Regression modelling for predictors of good functional outcome (modified Rankin scale 0–2 at 3 months)

	OR (95% CI)	Elimination step	*p* value
Age	0.96 (0.95–0.98)	/	< 0.001
ASPECT score	1.26 (1.09–1.46)	/	0.002
Systolic Blood Pressure on admission	0.99 (0.98–1)	/	0.054
NIHSS on admision	0.93 (0.89–0.97)	/	< 0.001
Thrombolysis	0.62 (0.39–1.1)	/	0.053
Previous ischemic stroke	1.03 (0.47–2.27)	1	0.936
CHA2DS2VASC score	1.02 (0.78–1.33)	2	0.906
Female	1.14 (0.73–1.76)	3	0.571
Anticoagulation	0.76 (0.39–1.48)	4	0.422
Hypertension	1.3 (0.83–2.05)	5	0.249
Smoking	0.41 (0.15–1.17)	6	0.095

**Table 5:** Regression modelling for predictors of successful reperfusion (mTICI 2b-3)

	OR (95% CI)	Elimination step	*p* value
Age	0.98 (0.96–1)	/	0.034
Female	0.64 (0.38–1.07)	/	0.085
Previous ischemic stroke	0.92 (0.36–2.34)	1	0.855
CHA2DS2VASc	1.03 (0.75–1.42)	2	0.850
Anticoagulation	0.81 (0.38–1.72)	3	0.582
Hypertension	1.25 (0.7–2.21)	4	0.454
ASPECT score	1.1 (0.92–1.3)	5	0.297
NIHSS	1.03 (0.98–1.08)	6	0.266
Thrombolysis	0.73 (0.42–1.24)	7	0.245
Systolic Blood Pressure on admission	0.99 (0.98–1)	8	0.120

## Discussion

Our prospective study based on a propensity-matched analysis showed that pre-admission anticoagulant use was not associated with an increased risk of symptomatic or any ICH in patients with ischaemic stroke and LVO of the anterior circulation treated with MT. Our findings are in line with most of the previous observational retrospective studies that did not document an increased risk of haemorrhage in patients on OAC treated with MT, except for Meinel [19] and Ramos [3] that observed an increased risk in studies conducted with large sample sizes. Our findings confirm the results of the study of Goldhoorn et al. (MR CLEAN Registry) in which the prior use of OAC in patients treated with endovascular treatment for ischemic stroke is not associated with an increased risk of SICH compared with no prior OAC use. However, in the study of Goldhoorn et al. only 19.52% were on DOACs while the remaining (80.48%) were on VKAs. Conversely in our study the majority of patients in the anticoagulation group (78.78%) were on DOACs and this difference might limit the comparison between the two studies. A recent pooled meta-analysis [4], performed on fifteen non-randomized studies, investigated the safety and efficacy of MT in acute ischemic stroke patients receiving anticoagulants (1281 on VKAs and 361 on DOACs). The authors concluded that patients on VKAs, but not patients on DOACs, had an increased risk of symptomatic ICH and mortality. However, it is noteworthy to mention that in most of the studies enrolled in this meta-analysis patients on treatment with VKAs did not have a significant higher risk of symptomatic ICH. Moreover, this meta-analysis carried several limitations as the heterogeneity of the groups of patients, the retrospective designs of the studies and differences in the outcome measurement tools. This can indicate that an incomplete adjustment of the confounding factors might have influenced the outcome of the analysis. Our analysis included 21 patients on VKAs and this limits the comparison with the results of the above-mentioned meta-analysis. Of note, to date, only our study and the study of Seiffge et al. [8]. used a propensity-matched analysis to adjust for possible confounding factors to investigate the safety of MT in patients on treatment with OAC compared to those without previous OAC. Both our data and Seiffge et al. analysis suggested that MT in patients on OAC is not associated with an increased risk of ICH. Although ICH could represent a major concern in anticoagulated patients undergoing MT, we believe that MT in OAC patients seems reasonably safe and the pre-admission anticoagulation status should not unnecessarily delay the procedure. Indeed, the percentage of symptomatic ICH in our OAC cohort was comparable to the rates of symptomatic ICH documented in literature [20, 21].

In our study, the 90-days postprocedural functional independence was achieved in 39.5% of the OAC patients and 33.7% of the controls. This difference resulted to be no-statistically significant, and our regression analysis showed that the anticoagulation status was not associated with functional independence at 90 days. Another important finding of our analysis is that we documented similar recanalization rates in our OAC and control arms, indicating that pre-admission anticoagulant use is not a predictor of successful reperfusion. Our data confirmed the results of the binary logistic regression analysis, made by Kupper et al. [22], of the German Stroke Registry-Endovascular Treatment on 1306 OAC patients and 4867 controls. This analysis revealed no influence of OAC status on good outcome at 3 months. Conversely, the meta-analysis by Chen et al. [4] illustrated poor functional outcome in patients receiving VKAs. However, it is possible to consider that the high mortality rate in the VKA subgroup of patients have contributed to the result. In our analysis, we observed that OAC patients experienced a low rate (1%) of sICH and we believe that this might represent a significant factor to possibly explain their reduced mortality rate. Overall, our analysis showed that MT in selected patients with ischemic stroke under OAC treatment did not raise any suggestion of safety and efficacy concerns at least in experienced stroke centres.

Our analysis had the following strengths: (1) data ascertainment was undertaken systematically and prospectively; (2) the use of a propensity-matched analysis to adjust for possible confounding factors. (3) large cohorts of patients as a single-centre study. Nevertheless, our study had several limitations. First, the non-randomized design of the study might have introduced bias. However, we opted to use a propensity-matched analysis to adjust for possible confounding factors. Moreover, information on functional outcome assessment at 90 days could be reached in 524 patients (90.8%). The study was conducted as single-centre study. We have included in our study 99 anticoagulated patients which represents still a relatively small size and raises the possibility that there may be small differences in outcomes between groups that might not have been detected. A significant difference in terms of use of IVT was also documented in our analysis where IVT was less frequently administered in patients on prior anticoagulant therapy (34% vs 84%, *P* < 0.01). Even though we adjusted for this factor in the logistic regression analyses to determine their impact on the outcomes this could represent a potential bias. Another possible limitation of our work is that we did not report the stroke etiology as a variable in the propensity score analysis. This is because over 60% of the patients that are treated at our thrombectomy centre are referred from the primary stroke centres. These patients are normally transferred back to the referring centre after 24 h after the procedure and therefore we are not able to perform in these patients the basic investigations to establish the etiology of their stroke.

## Conclusions

In conclusion, our analysis confirmed previous observations showing that mechanical thrombectomy is reasonably safe in patients on treatment with OAC and the pre-admission anticoagulation status should not unnecessarily delay the procedure. For this reason, in-hospital protocols incorporating anticoagulation status are needed to optimize acute stroke management from the door of the emergency department to the angiographic room, requiring multi-disciplinary groups to work together. However, confirmation of these results in larger controlled prospective cohorts is necessary.

### Supplementary Information

Below is the link to the electronic supplementary material.

Supplementary file1 (DOCX 40 KB)

## Data Availability

Data are available upon reasonable request.
