# Supplementary material for: Outcomes of mechanical thrombectomy in orally anticoagulated patients with anterior circulation large vessel occlusion: a propensity-matched analysis of the Imperial College Thrombectomy Registry
Source: J Neurol. 2023 Aug 18;270(12):5827–34. doi: 10.1007/s00415-023-11926-5 (PMC10632297; doi:10.1007/s00415-023-11926-5)
Supplement: Supplementary file 1 — Supplementary file1 (DOCX 40 KB) [file 415_2023_11926_MOESM1_ESM.docx]

| **Supplementary Table 1** – Cohort characteristics before Propensity score match analysis | | |
| --- | --- | --- |
|  | **No anticoagulation** | **Anticoagulation** |
| n | 474 | 99 |
| Age | 66 ± 14.8 | 72 ± 11.1** |
| CHA2DS2VASc | 4 (4-5) | 5 (5-6)*** |
| Female | 210(44.3%) | 52(52.5%) |
| Hypertension | 257(54.2%) | 65(65.7%)* |
| Diabetes | 90(19%) | 21(21.2%) |
| Dyslipidemia | 216(45.6%) | 46(46.5%) |
| CAD | 76(16%) | 19(19.2%) |
| Non-smoking | 371(78.3%) | 83(83.8%) |
| Previous ischemic stroke | 41(8.6%) | 25(25.3%)*** |
| Malignancy | 39(8.2%) | 13(13.1%) |
| Dementia | 4(0.8%) | 0(0%) |
| mRS 0-2 at baseline | 473(100%) | 99(100%) |
| Hemoglobin | 131 ± 20.8 | 127.4 ± 20.1 |
| Platelets (x10^9/L) | 232.9 ± 153.8 | 221.6 ± 94.5 |
| aPTT | 30.6 ± 15.4 | 35.4 ± 31.6** |
| Creatinine | 81.5 ± 53.4 | 82.9 ± 28.4 |
| Systolic BP | 144 ± 24.1 | 146.6 ± 26.5 |
| Diastolic BP | 80.6 ± 15.5 | 82.4 ± 16.3 |
| Thrombolysis | 391(82.5%) | 22(22.2%)*** |
| NIHSS at baseline | 16.9 ± 5.8 | 18 ± 5.1 |
| Onset to needle (mins) | 130 ± 54.5 | 136 ± 56.9 |
| Door to needle (mins) | 42 ± 28.9 | 55 ± 42 |
| Onset to groin (mins) | 280 ± 89 | 297 ± 170.4 |
| ASPECTS | 8 ± 1.5 | 8 ± 1.4 |
| *p<.05, **p<.01, ***p<0.001 |  |  |

**Supplementary Table 2. Endovascular therapy techniques.**

| **Endovascular therapy [n, (%)]** | OAC n=99 (%) | No-OAC n=396 (%) | P = 0.851 |
| --- | --- | --- | --- |
| Stent retriever | 19 (19) | 79 (20) |  |
| Thromboaspiration | 49 (50) | 205 (51.8) |  |
| Thromboaspiration + Stent retriever | 31 (31) | 112 (28.2) |  |

**Supplementary Figure 1** – Standardized differences in variables included in the propensity score

**
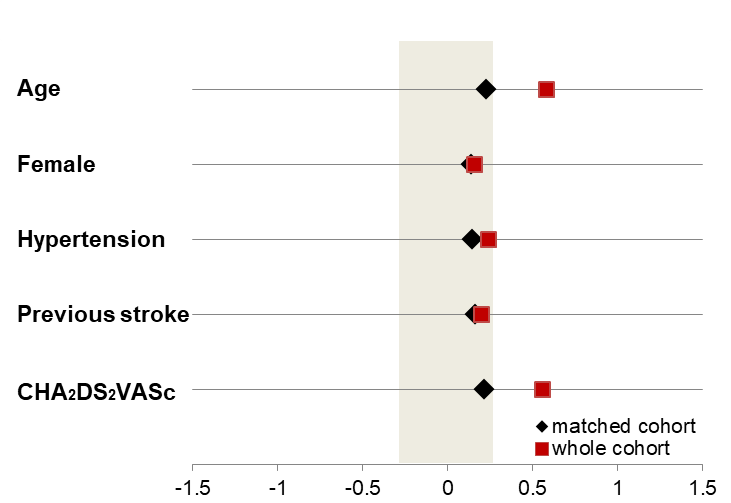
**

**Supplementary Table 3 –** Distribution of risk factors for good functional outcome (mRS 0-2 at 90 days)

|  | **mRS >2** | **mRS 0-2** |
| --- | --- | --- |
| n | 295 | 183 |
| Age | 73 ± 12.1 | 66 ± 12.8*** |
| CHA2DS2VASc | 5 (5-6) | 4 (4-5)** |
| Anticoagulation | 65 (22%) | 33 (18%) |
| Female | 142 (48.1%) | 83 (45.4%) |
| Hypertension | 194 (65.8%) | 92 (50.3%) |
| Diabetes | 72 (24.4%) | 34 (18.6%) |
| Dyslipidemia | 138 (46.8%) | 101 (55.2%) |
| CAD | 64 (21.7%) | 27 (14.8%) |
| Non-smoking | 246 (83.4%) | 138 (75.4%)* |
| Previous stroke | 26 (8.8%) | 15 (8.2%) |
| Malignancy | 31 (10.5%) | 15 (8.2%) |
| Dementia | 4 (1.4%) | 0 (0%) |
| mRS baseline 0-2 | 295 (100%) | 182 (100%) |
| PLT (*10^9/L) | 226.5 ± 91.2 | 245.2 ± 220.4 |
| APTT (secs) | 31.4 ± 19.3 | 32.5 ± 22.7 |
| INR | 6.7 ± 25.5 | 1.8 ± 4.4 |
| Creatinine (umol/l) | 87.1 ± 66.2 | 78.3 ± 24.7 |
| Systolic BP (mmHg) | 149 ± 24 | 141 ± 24.9*** |
| Diastolic BP (mmHg) | 81 ± 15.1 | 80 ± 16.8 |
| Thrombolysis | 196 (66.4%) | 140 (76.5%)* |
| NIHSS on presentation | 18 ± 5.4 | 16 ± 5.6*** |
| ONT (minutes) | 131 ± 51.1 | 128 ± 61.8 |
| DNT (minutes) | 41 ± 26.9 | 44 ± 35 |
| OGT (minutes) | 290 ± 114.6 | 269 ± 96.3 |
| ASPECT score | 8 ± 1.5 | 8 ± 1.4*** |
| Successful recanalization (TICI 2b-3) | 225 (77.1%) | 165 (92.2%)*** |
| sICH | 21 (7.1%) | 0 (0%)*** |
| Any ICH | 74 (25.1%) | 20 (10.9%)*** |

*p<.05, **p<.01, ***p<0.001
